# Molecular alterations in colorectal adenomas and intramucosal adenocarcinomas defined by high-density single-nucleotide polymorphism arrays

**DOI:** 10.1007/s00535-017-1317-2

**Published:** 2017-02-14

**Authors:** Makoto Eizuka, Tamotsu Sugai, Wataru Habano, Noriyuki Uesugi, Yayoi Takahashi, Keisuke Kawasaki, Eiichiro Yamamoto, Hiromu Suzuki, Takayuki Matsumoto

**Affiliations:** 1 Department of Molecular Diagnostic Pathology, School of Medicine, Iwate Medical University, 19-1, Uchimaru, Morioka, 020-8505 Japan; 2 Department of Pharmacodynamics and Molecular Genetics, School of Pharmacy, Iwate Medical University, Morioka, Japan; 3 Division of Gastroenterology, Department of Internal Medicine, Iwate Medical University, Morioka, Japan; 4 Department of Molecular Biology, School of Medicine, Sapporo Medical University, Sapporo, Japan

**Keywords:** Colorectal adenoma, Copy number alteration, Intramucosal adenocarcinoma, Gain, Loss of heterozygosity

## Abstract

**Background:**

We examined colorectal adenomas and intramucosal adenocarcinomas (IMAs) to develop a genome-wide overview of copy number alterations (CNAs) during colorectal tumorigenesis.

**Methods:**

We analysed CNAs using a high-resolution SNP array of isolated tumour glands obtained from 55 colorectal adenomas (35 low-grade adenomas and 20 high-grade adenomas) and 30 IMAs. Next, we examined whether frequent CNAs differed between low-grade and high-grade adenomas or high-grade adenomas and IMAs. Finally, we investigated the total lengths of the CNAs in low-grade adenomas, high-grade adenomas, and IMAs.

**Results:**

Although no frequent CNAs were found in low-grade adenomas, the most frequent alterations of high-grade adenomas were gains of 7q11, 7q21 and 9p13 and loss of 5q14.3-35. High levels of gains were detected at 13q, 7q, 8p, 20q, 7p, 18p and 17p in IMAs. Although no frequent alteration differed between low-grade and high-grade adenomas, significant differences of gains at 13q, 17p and 18p were found between high-grade adenoma and IMAs. Although the total lengths of all CNAs (gains and losses), copy number gains, and losses of heterozygosity were significantly greater in high-grade adenomas than in low-grade adenomas, no significant differences in the lengths of CNAs were found between high-grade adenomas and IMAs.

**Conclusions:**

Genomic alterations play an essential role in early colorectal carcinogenesis. CNAs in colorectal tumours provide new insights for evaluation of colorectal tumorigenesis.

**Electronic supplementary material:**

The online version of this article (doi:10.1007/s00535-017-1317-2) contains supplementary material, which is available to authorized users.

## Introduction

Although there have been advances in medical treatment of colorectal cancer (CRC) recently, CRC remains a major cause of cancer death in both men and women worldwide [1]. Colorectal adenoma is a well-known premalignant lesion in colorectal carcinogenesis. Intramucosal cancer is an intermediate lesion between colorectal adenoma and invasive submucosal cancer, which is associated with a high risk of metastasis [2]. If intramucosal cancer is left untreated, it would eventually invade the submucosal layer [2]. Colorectal adenoma and intramucosal cancer (adenocarcinoma) are important lesions for our understanding of early colorectal carcinogenesis [3].

The sequential acquisition of molecular alterations within the adenoma–carcinoma progression is an important theory in the molecular carcinogenesis of CRC [4–6]. Fearon and Vogelstein first proposed the multistep genetic model of colorectal carcinogenesis that is now a paradigm for CRC progression. Inactivation of the adenomatous polyposis coli tumour-suppressor gene (*APC*) occurs first, followed by activating mutations of *KRAS*. Next, inactivation of the *TP53* gene occurs at the intramucosal cancer stage. Finally, multiple loss-of-heterozygosity (LOH) events accumulate in the invasive stage of CRC [4–6]. Although recent studies have shown that DNA methylation is closely associated with the initial development of CRC [7, 8], genomic alterations are required to achieve invasive ability in the current molecular pathways [6–8]. Advanced array technology has made possible identification of genome-wide alterations in colorectal carcinogenesis. Genomic alterations can be classified into subtypes: copy number gains and copy number losses (both LOH and copy-neutral LOH) [9–11]. Although copy number alterations (CNAs) have been extensively investigated in CRC [12–17], the role of CNAs is not fully understood in the initial stages of colorectal carcinogenesis, particularly colorectal adenoma and intramucosal adenocarcinoma [16, 17].

In this study, we mapped the overall genetic changes in colorectal adenomas (low grade and high grade) and intramucosal adenocarcinomas using high-resolution single-nucleotide polymorphism (SNP) mapping arrays. Our goal was to search for differences in frequent genetic alterations between colorectal adenoma and intramucosal adenocarcinoma samples that may identify candidate genes highly characteristic of malignant transformation from colorectal adenoma. In addition, we aimed to identify genetic alterations between low-grade and high-grade adenomas.

## Materials and methods

### Patients

Eighty-five samples were obtained from patients with newly diagnosed colorectal adenoma (55 cases) and intramucosal adenocarcinoma (30 cases) at Iwate Medical University Hospital between December 2014 and August 2016. The colorectal adenomas consisted of 35 low-grade and 20 high-grade cases. Histological classification was performed according to the modified criteria of the WHO classification for colorectal tumours [18]. Intramucosal adenocarcinoma was classified as well-differentiated adenocarcinoma (21 cases) or moderately differentiated adenocarcinoma (9 cases). Poorly differentiated adenocarcinoma was excluded. Clinicopathology data are listed in Table 1. The local ethics committees of Iwate Medical University approved the sample collection and study design. General written consent was obtained from all patients.

**Table 1:** Clinicopathology data for the colorectal adenomas and intramucosal adenocarcinomas (*IMAs*)

	Adenomas	IMAs
Total	55	30
Male	36	21
Female	19	9
Age (years)^a^	67.0 (42–90)	66.5 (48–88)
Locus		
Right (C, A, T)	20 (36.4%)	9 (30.0%)
Left (D, S, R)	35 (63.6%)	21 (70.0%)
Size (mm)^a^	14.0 (7-53)	15.0 (9-41)
Macroscopic type		
Is	3 (5.5%)	6 (20.0%)
Isp	21 (38.2%)	8 (26.7%)
Ip	24 (43.6%)	3 (10.0%)
IIa	1 (1.8%)	1 (3.3%)
IIc	0	5 (16.7%)
LST	6 (10.9%)	7 (23.3%)
Adenoma component		
TA	37 (67.3%)	
TVA	18 (32.7%)	
Tumour grade		
Low	35 (63.6%)	
High	20 (36.4%)	
Differentiation		
tub1		21 (70.0%)
tub2		9 (30.0%)

*C* cecum, *A* ascending colon, *T* transverse colon, *D* descending colon, *S* sigmoid colon, *R* rectum, *LST* laterally spreading tumour, *TA* tubular adenoma, *tub1* well-differentiated adenocarcinoma, *tub2* moderately differentiated adenocarcinoma, *TVA* tubulovillous adenoma

^a^The median is given, with the range in *parentheses*

All samples were processed by the Diagnostic Pathology Laboratory of Iwate Medical University Hospital. Samples were routinely fixed in 10% neutral-buffered formalin, processed, paraffin-embedded, step and serial sectioned, and stained with haematoxylin and eosin after sampling for molecular analysis.

### Grades of atypia (dysplasia) of colorectal adenoma and definition of intramucosal adenocarcinoma

On the basis of the degree of cytological atypia and abnormal crowding of the epithelium, colorectal adenoma is classified into two categories: low-grade and high-grade colorectal adenoma [18]. Low-grade adenoma is characterized by a uniform monolayer of columnar cells with basal nuclei showing minimal atypia [1]. High-grade adenoma has greater nuclear atypia, with nuclear pleomorphism, nuclear enlargement and pseudostratification. Intramucosal adenocarcinoma has marked cytological atypia and a complex architecture with cribriform groups, irregular branching and budding of neoplastic cells into the lumen [18]. It is common to observe different grades of atypia (dysplasia) within a given lesion, which suggests the development of atypia (dysplasia) from a lower grade to a higher grade. Distinguishing the grade of atypia (dysplasia) is important, as invasive carcinoma is commonly observed in areas of high-grade atypia (dysplasia). In each case, the lesion should be classified according to the highest grade of dysplasia observed. Sections of colorectal adenomas and intramucosal adenocarcinomas were reviewed and analysed by a senior gastrointestinal pathologist blinded to patient outcomes. The slides were independently evaluated by experienced pathologists (T.S. and N.U.). In cases in which the initial evaluation provided different results, a consensus interpretation was reached after reexamination. Additionally, a consensus diagnosis was obtained in cases in which different results were obtained for initial routine histological diagnoses.

### Crypt isolation and DNA extraction

Crypts were isolated from tumour and normal mucosa samples to obtain pure glands in accordance with a previously reported method [19]. The isolated glands were routinely processed to confirm the nature of the glands by means of paraffin-embedded histological sections. Contamination with other materials such as interstitial cells was not evident in the samples that were examined, as described in previous reports [19, 20].

DNA from normal and tumour glands was extracted by standard sodium dodecyl sulfate–proteinase K treatment. DNA extracted from the samples was resuspended in a buffer of 10 mM tris(hydroxymethyl)aminomethane–HCl and 1 mM EDTA, pH 8.0.

### CNA analysis

Analysis of CNAs was performed according to our previous reports [21, 22]. Extracted DNA was adjusted to a concentration of 50 ng/μL. All 85 paired samples were assayed with use of the Infinium HumanCytoSNP-12 v2.1 BeadChip (Illumina, San Diego, CA, USA), which contains 299,140 SNP loci, according to the Illumina Infinium HD assay protocol. BeadChips were scanned by iScan (Illumina) and analysed by GenomeStudio (version 2011.1; Illumina). The log *R* ratio (LRR) and B allele frequency (BAF) for each sample were exported from normalized Illumina data with use of GenomeStudio. Data were analysed with use of KaryoStudio 1.4.3 (CNV Plugin version 3.0.7.0; Illumina) with default parameters. Chromosomal CNAs were classified by copy number variation partition algorithms: LRR = 0 indicated a normal diploid region, LRR > 0 indicated a copy number gain and LRR < 0 indicated a copy number LOH. BAFs ranged from 0 to 1; homozygous SNPs had BAFs near 0 (A allele) or 1 (B allele), and heterozygous diploid region SNPs had BAFs near 0.5 (AB genotype). Additionally, LRR and BAF data were used to identify regions of hemizygous and copy-neutral LOH.

### Calculation of CNA length on a genome-wide scale in CRCs

To quantify CNAs on a genome-wide scale, we calculated the total lengths of CNAs (losses plus gains), total length of copy number gains, total length of copy number LOH events and total length of copy-neutral LOH events identified by the SNP array analysis, as previously described [21, 22]. We used the total CNA length as an index representing the degree of chromosomal alterations and assessed the relationship between CNA length (total CNA, gain, LOH and copy-neutral LOH) and low-grade adenoma, high-grade adenoma or intramucosal adenocarcinoma.

### Statistical analysis

Data obtained for CNAs based on each subgroup were analysed by chi-square tests with Yates’s correction with the aid of Stat Mate-III (Atom, Tokyo, Japan). In addition, differences in the total lengths of CNAs between the three groups were analysed by Kruskal–Wallis tests (Prism 6; GraphPad Software, La Jolla, CA, USA). If statistical differences between the three groups were found, statistical analysis of the two groups was further performed by Mann–Whitney *U* tests (Prism 6) with Bonferroni corrections. Differences with *P* < 0.05 were regarded as significant.

## Results

### Genomic alterations in colorectal adenomas and intramucosal adenocarcinomas

**Fig. 1:**
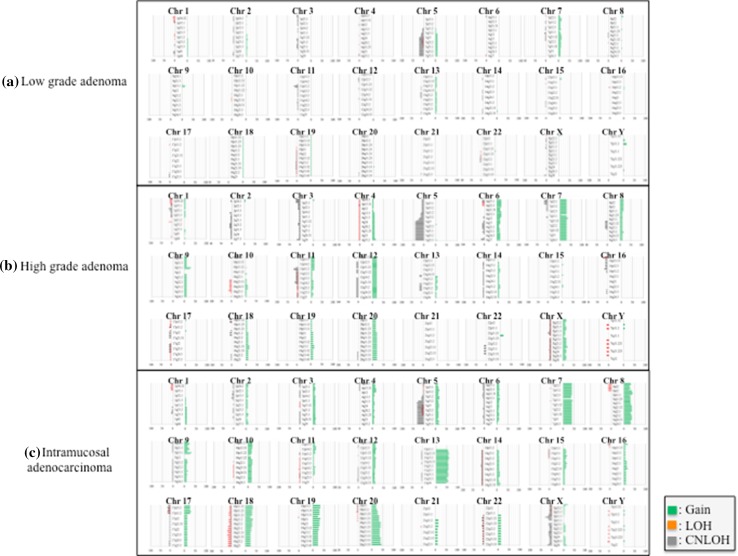
Ideograms of genomic imbalance in 85 colorectal tumours comprising low-grade adenoma, high-grade adenoma or intramucosal adenocarcinoma. Chromosomes are ordered from 1 to 22. The *coloured horizontal lines* represent the frequencies of gains, loss of heterozygosity (*LOH*) and copy-neutral LOH (*CNLOH*). *Lines on the left* indicate losses (*red* LOH; *grey* CNLOH), and *lines on the right* (*green*) indicate gains

The 85 pairs of colorectal adenoma and intramucosal adenocarcinoma samples were examined with use of SNP arrays to detect CNAs in colorectal adenomas (low-grade and high-grade adenomas) and intramucosal adenocarcinomas (Fig. 1). The frequency of CNAs was detected across the entire genome. In colorectal adenomas, the mean total number of chromosomal aberrations per patient was 59, with the number of gains averaging 32 and ranging from 0 to 163, the number of LOHs averaging 5 and ranging from 0 to 83, and the number of copy-neutral LOHs averaging 22 and ranging from 0 to 153. Next, we examined genomic alterations on the basis of the grade of atypia (dysplasia) in colorectal adenomas. In low-grade colorectal adenomas, the mean number of total chromosomal aberrations was 28 per patient, with the number of gains averaging 13 and ranging from 0 to 45, the number of LOHs averaging 3 and ranging from 0 to 21, and the number of copy-neutral LOHs averaging 12 and ranging from 0 to 68. In high-grade colorectal adenomas, the mean number of total chromosomal aberrations per patient was 113, with the number of gains averaging 64 and ranging from 0 to 163, the number of LOHs averaging 11 and ranging from 0 to 83, and the number of copy-neutral LOHs averaging 38 and ranging from 0 to 153. In intramucosal adenocarcinomas, on the other hand, the mean number of total chromosomal aberrations per patient was 168, with the number of gains averaging 126 and ranging from 0 to 357, the number of LOHs averaging 11 and ranging from 0 to 67, and the number of copy-neutral LOHs averaging 31 and ranging from 0 to 132. We searched for minimal common CNA regions in colorectal adenomas and intramucosal adenocarcinomas but could not find minimal common CNA regions in more than 30% of cases in the tumours examined.

Although none of the CNAs found in more than 30% of cases were detected in low-grade colorectal adenomas, regions of gain located at 7q11.22-23, 7q11.1-11.21, 7q21.11-36.3, 7p11.2-22.3 and 9p13.1 (in decreasing order of frequency) were detected in more than 30% of high-grade colorectal adenomas. Regions of copy-neutral LOH detected in more than 30% of cases were found at 5q15-35.3 and 5q14.3 in high-grade colorectal adenomas. LOH was not detected in either low-grade or high-grade colorectal adenomas. In intramucosal adenocarcinomas, regions of gain defined in more than 30% of cases were located at 13q12.13-12.2, 13q13.2-13.3, 13q31.1-32.1, 13q33.2-3, 13q21.1-22.1, 13q14.11-14.13, 13q14.3, 13q12.11-12.12, 13q12.3-13.1, 13q11, 13q14.2, 13q22.2-33.1, 13q32.2-32.3, 13q34, 7q11.21, 20q13.33, 7p12.3-22.3, 7q11.22-36.3, 8p11.21-11.22, 20q11.21-13.32, 7p11.2-12.2, 8q21.11-24.13, 20q12-13.13, 18p11.31-32, 17p12 and 18q12.1-12.3. Whereas regions of copy-neutral LOH detected in more than 30% of cases were found at 5q14.1 and 5q14.2-35.3 in intramucosal adenocarcinomas, no LOH events were found in intramucosal adenocarcinomas. These results are summarized in Table 2.

**Table 2:** Frequent copy number alteration regions in colorectal adenomas and intramucosal adenocarcinoma (*IMA*)

Chromosomal regions	LGA (*n* = 35)	Chromosomal regions	HGA (*n* = 20)	Chromosomal regions	IMA (*n* = 30)
Gain		Gain		Gain	
None		7q11.22-23	7 (35.0%)	13q12.13-12.2, q13.2-13.3	18 (60.0%)
CNLOH		7q11.1-11.21	6 (30.0%)	13q31.1-32.1, q33.2-3	17 (56.7%)
None		7q21.11-36.3	6 (30.0%)	13q21.1-22.1	17 (56.7%)
LOH		7p11.2-22.3	6 (30.0%)	13q14.11-14.13, q14.3	17 (56.7%)
None		9p13.1	6 (30.0%)	13q12.11-12.12, q12.3-13.1	17 (56.7%)
		CNLOH		13q11, q14.2, q22.2-33.1	16 (53.3%)
		5q15-35.3	8 (40.0%)	13q32.2-32.3	15 (50.0%)
		5q14.3	7 (35.0%)	13q34	14 (46.7%)
		LOH		7q11.21, 20q13.33	13 (43.3%)
		None		7p12.3-22.3, 7q11.22-36.3	12 (40.0%)
				8p11.21-11.22	12 (40.0%)
				20q11.21-13.32	12 (40.0%)
				7p11.2-12.2, 8q21.11-24.13	11 (36.7%)
				20q12-13.13	11 (36.7%)
				18p11.31-32	10 (33.3%)
				17p12, 18q12.1-12.3	9 (30.0%)
				CNLOH	
				5q14.1	10 (33.3%)
				5q14.2-35.3	9 (30.0%)
				LOH	
				None	

*CNLOH* copy-neutral loss of heterozygosity, *HGA* high-grade adenoma, *LGA* low-grade adenoma, *LOH* loss of heterozygosity

### Genomic differences between low-grade and high-grade colorectal adenomas and between high-grade colorectal adenomas and intramucosal adenocarcinomas

Next, we examined differences in CNAs between low-grade and high-grade colorectal adenomas and between high-grade colorectal adenomas and intramucosal adenocarcinomas. We compared regions of gain detected in more than 30% of cases between low-grade colorectal adenomas and high-grade colorectal adenomas. Significant differences in gains between low-grade and high-grade colorectal adenomas were found at 7p22.2-22.3. However, no significant differences in the frequencies of copy-neutral LOH or LOH were found between low-grade and high-grade colorectal adenomas.

We also compared the regions of gain detected in more than 30% of cases between high-grade colorectal adenomas and intramucosal adenocarcinomas. Significant differences in gains between high-grade colorectal adenomas and intramucosal adenocarcinomas were found at 13q12.13-2, 13q13.2-3, 13q12.11-13.1, 13q14.11-14.13, 13q11, 13q14.2, 13q32.2, 13q21.2, 13q22.1, 13q32.1, 13q33.2-3, 13q22.2-33.1, 13q14.3-21.1, 13q21.31-33, 13q31.1-3, 13q32.3, 13q34, 17p12, 17p11.2, 17p13.1-13.2 and 18p11.31-32 (Table 3). However, differences in regions of copy-neutral LOH and LOH were not detected between high-grade colorectal adenomas and intramucosal adenocarcinomas.

**Table 3:** Significant differences in the frequencies of copy number alterations between high-grade colorectal adenoma (*HGA*) and intramucosal adenocarcinoma (*IMA*)

Chromosomal regions	HGA (*n* = 20)	IMA (*n* = 30)	*P*
Gain			
13q12.13-2, 13q13.2-3	0	18 (60.0%)	<0.001
13q12.11-13.1, 13q14.11-14.13	0	17 (56.7%)	<0.001
13q11, 13q14.2	0	16 (53.3%)	<0.001
13q32.2	0	15 (50.0%)	<0.001
13q21.2, 13q22.1, 13q32.1, 13q33.2-3	1 (5.0%)	17 (56.7%)	<0.01
13q22.2-33.1	1 (5.0%)	16 (53.3%)	<0.01
13q14.3-21.1, 13q21.31-33, 13q31.1-3	2 (10.0%)	17 (56.7%)	<0.01
13q32.3	1 (5.0%)	15 (50.0%)	<0.01
13q34	1 (5.0%)	14 (46.7%)	<0.01
17p12	0	9 (30.0%)	0.02
17p11.2, 17p13.1-13.2	0	8 (26.7%)	0.03
18p11.31-32	1 (5.0%)	10 (33.3%)	0.04
CNLOH			
None			
LOH			
None			

*CNLOH* copy-neutral loss of heterozygosity, *LOH* loss of heterozygosity

### Difference in CNA length on a genome-wide scale in colorectal adenomas and intramucosal adenocarcinomas

**Fig. 2:**
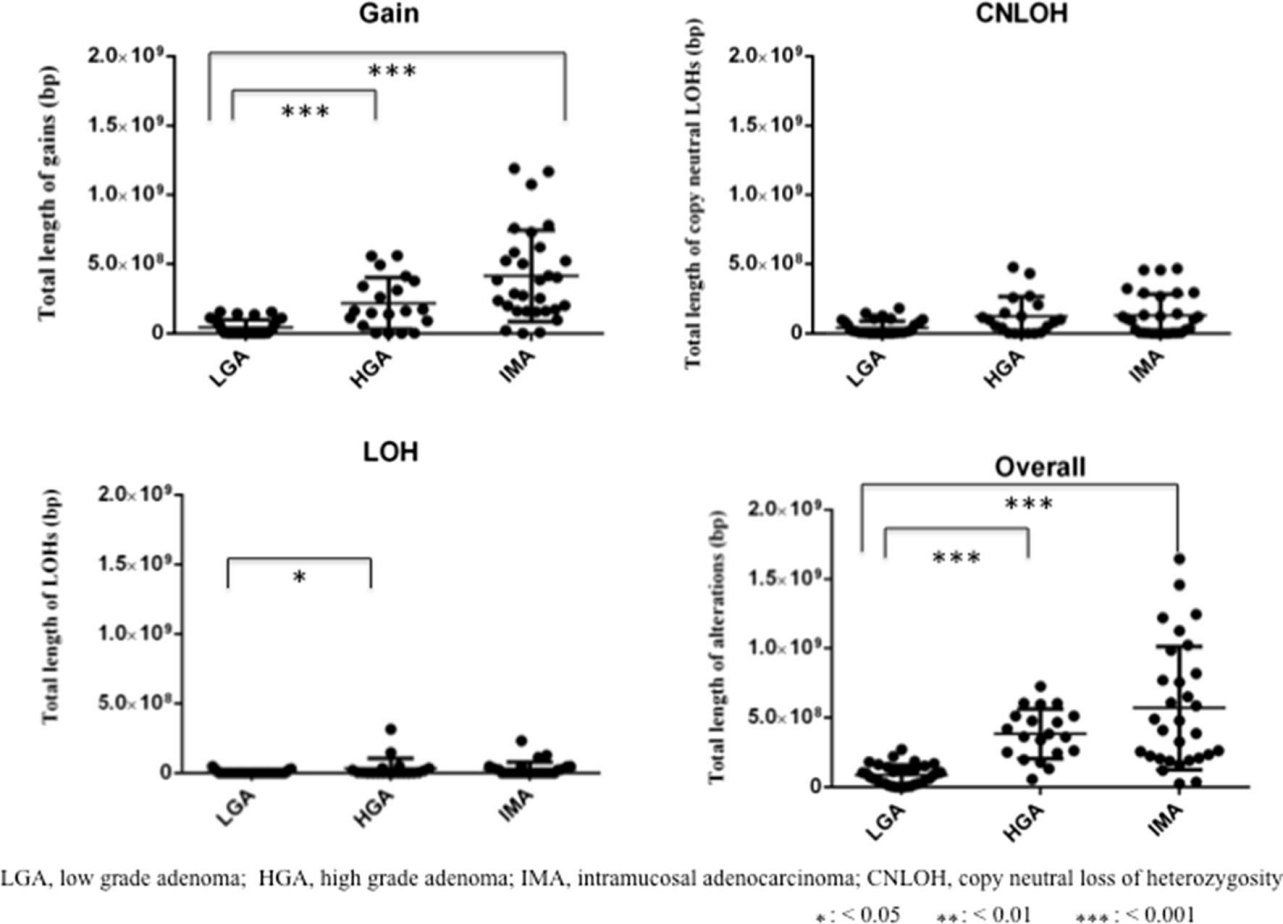
Comparison of the total lengths of abnormal regions containing copy number alterations between patients with low-grade adenoma (*LGA*), high-grade adenoma (*HGA*), or intramucosal adenocarcinoma (*IMA*). *CNLOH* copy-neutral loss of heterozygosity, *LOH* loss of heterozygosity, *one asterisk* *P* < 0.05, *two asterisks* *P* < 0.01, *three asterisks* *P* < 0.001

Overall, the total length of CNAs was greater in high-grade colorectal adenoma than in low-grade colorectal adenoma (Fig. 2; *P* < 0.001) and greater in intramucosal adenocarcinoma than in low-grade colorectal adenoma. Genomic losses (LOH and copy-neutral LOH) and gains were investigated separately. The total length of copy number gains was significantly greater in high-grade colorectal adenoma than in low-grade colorectal adenoma (Fig. 2; *P* < 0.001) and significantly greater in intramucosal adenocarcinoma than in low-grade colorectal adenoma (Fig. 2; *P* < 0.001). Although there was a significant difference in the total length of LOH between low-grade and high-grade colorectal adenoma (Fig. 2; *P* < 0.05), no differences in copy-neutral LOH between low-grade colorectal adenoma and high-grade colorectal adenoma were detected. No significant differences in the total length of losses (LOH and copy-neutral LOH) were found between high-grade colorectal adenoma and intramucosal adenocarcinoma.

### Genomic differences in low-grade colorectal adenomas, high-grade colorectal adenomas and intramucosal adenocarcinomas between left-sided and right-sided tumours

We examined CNAs of low-grade adenomas, high-grade adenomas, and intramucosal adenocarcinomas for left-sided and right-sided tumours. However, there were no significant differences in the frequencies of CNAs between left-sided and right-sided tumours in low-grade adenomas, high-grade adenomas, and intramucosal adenocarcinomas. These data are shown in Table S1.

### Genomic differences in low-grade colorectal adenomas, high-grade colorectal adenomas and intramucosal adenocarcinomas between rectal and colonic tumours

Next, we examined CNAs in low-grade adenomas, high-grade adenomas and intramucosal adenocarcinomas of the rectum and the colon. Although there were no differences in the frequencies of CNAs between the rectum and the colon for low-grade adenomas, a significant difference in the frequencies of CNAs between the rectum and the colon was observed for high-grade adenomas (gains at 8q23.2-3, 8p11.1 and p11.21-23.3). In addition, there were significant differences in the frequencies of CNAs between the rectum and the colon for intramucosal adenocarcinomas (greater for the rectum than for the colon: gains at 16q22.1-24.3, 21q21.3-22.3, 10q11.21-23, 10p11.1, 12q13.13-15, q21.2, q24.32, 12p11.1-13.31, 16q11.2-13 and 21q11.2-21.2; LOH at 16p13.3). The detailed results are shown in Table S2.

**Fig. 3:**
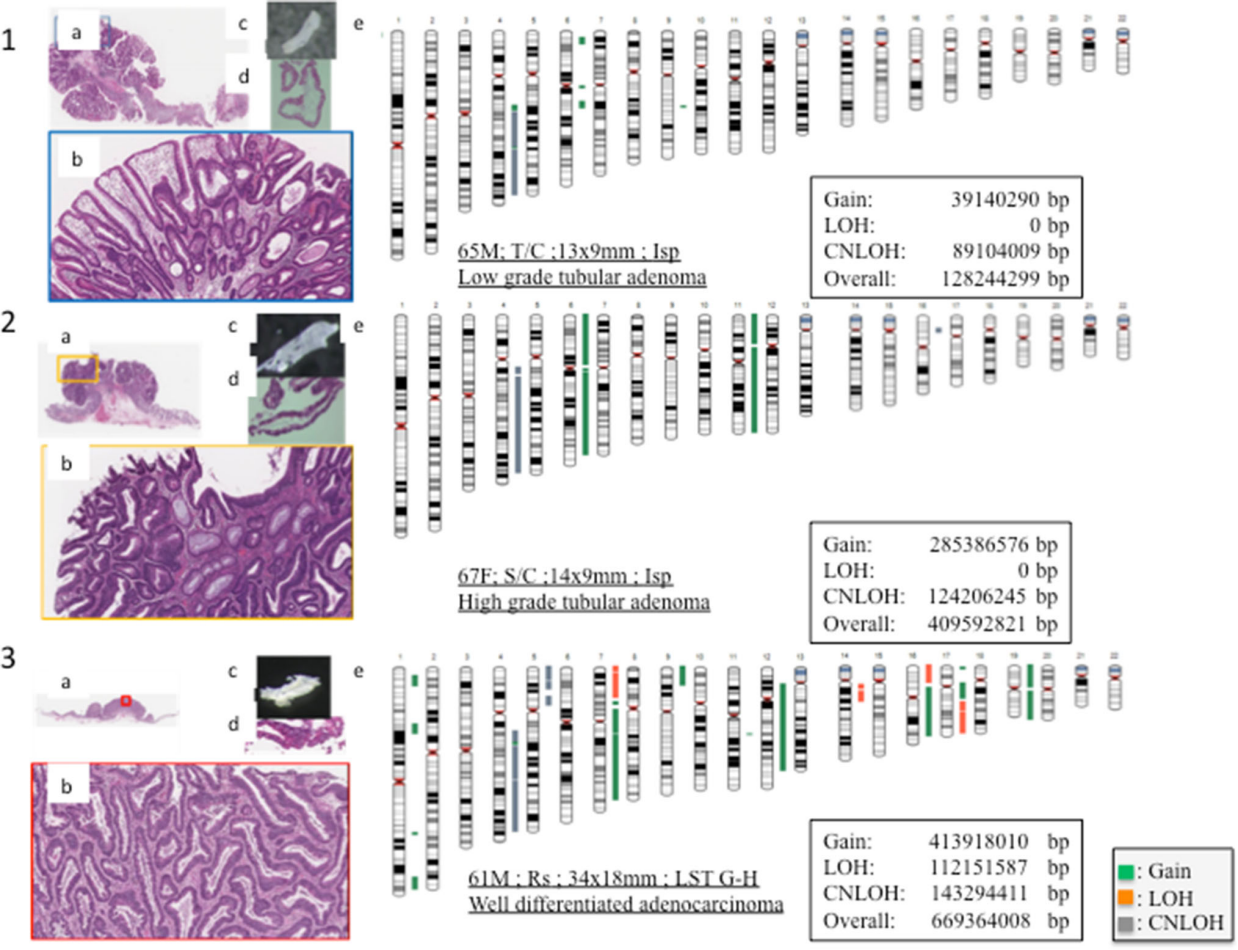
*1*: Representative images of low-grade adenoma (*a* loupe image of low-grade adenoma, *b* low-power view of the lesion showing low-grade adenoma, *c* an isolated tumour gland under a dissection microscope, *d* high-power histological view of the isolated gland) and an ideogram showing copy number variations (*e*). *2*: Representative images of high-grade adenoma (*a* loupe image of high-grade adenoma, *b* low-power view of the lesion revealing high-grade adenoma, *c* an isolated tumour gland under a dissection microscope, *d* high-power histological view of the isolated gland) and an ideogram showing copy number variations (*e*). *3*: Representative images of intramucosal adenocarcinoma (*a* loupe image of intramucosal adenocarcinoma, *b* low-power view of the lesion showing intramucosal adenocarcinoma, *c* an isolated tumour gland under a dissection microscope, *d* high-power histological view of the isolated gland) and an ideogram showing copy number variations (*e*). In the ideograms, *green* represents gain, *red* represents loss of heterozygosity (*LOH*) and *grey* represents copy-neutral loss of heterozygosity (*CNLOH*). *LST* laterally spreading tumour

Representative features of the pathological and molecular findings in colorectal adenoma (low grade and high grade) and intramucosal adenocarcinoma are shown in Fig. 3.

## Discussion

This study focused on the comprehensive genetic characterization of colorectal tumours occurring in the progression of colorectal adenoma to colorectal intramucosal adenocarcinoma. Recent studies have shown that DNA methylation is one of the major epigenetic mechanisms closely associated with development of low-grade colorectal adenoma [6, 7]. Although epigenetic alterations play an essential role in the early progression of CRC [23], genomic changes are also closely associated with the progression of CRC [6]. Genomic changes are classified into two groups: genomic gains and losses. In addition, genomic losses can be subclassified into two subgroups (i.e., LOH and copy-neutral LOH) with use of an SNP array. LOH and copy-neutral LOH are common mechanisms of inactivating the expression of tumour-suppressor genes [9]. To the best of our knowledge, this is the first study that has systematically used high-resolution SNP arrays to extensively compare genetic abnormalities found in colorectal adenomas and intramucosal adenocarcinomas. The present results may be of great clinical and pathological utility for the identification of patients with colorectal tumours at higher risk of tumour progression.

Contamination of tumour tissues with interstitial cells makes it difficult to accurately examine molecular alterations [19, 20]. In the present study, we used the crypt isolation method to analyse only the tumour epithelial component in the tumour tissue. Our previous reports have emphasized that isolation of the tumour glands is indispensable for evaluation of molecular alterations in gland-forming tumours [3, 19, 20]. In the present study, we successfully isolated tumour glands from all tumour tissues. Therefore, we could provide a reliable molecular analysis for understanding colorectal carcinogenesis using high-density SNP arrays [3, 19, 20].

In the present study, although no CNAs (gains and losses) detected in more than 30% of low-grade colorectal adenomas were observed, we identified frequently altered chromosomal sites in high-grade colorectal adenomas (gains at 7q11-36, 7p11 and 9p13, and copy-neutral LOH at 5q14-35). This finding suggests that accumulation of CNAs may not be necessary for the development of low-grade colorectal adenoma. On the basis of this evidence, we suggest that although genetic (chromosomal) and epigenetic alterations contribute to the pathogenesis of CRC, epigenetic alterations rather than chromosomal alterations are more important for development of low-grade colorectal adenoma [6, 7].

Multiple CNAs characterize molecular alteration of CRC according to previous reports, which have shown that accumulation of CNAs is necessary for progression of CRC [6, 7, 24]. The present study identified a number of focal genomic gains (at 13q12, 13q13, 13q31, 13q33, 13q21, 13q14, 13q12, 13q11, 13q14, 13q22, 13q32, 13q34, 7q11, 20q13, 7p12-22, 7q12-36, 8p11, 20q11-13, 7p11-12, 8q21-24, 20q12-13, 18p11, 17p12 and 18q12) and losses (copy-neutral LOH at 5q14-35) in colorectal intramucosal adenocarcinoma. These findings showed some concordance with the results from previous studies of CRC [16, 17]. However, to the best of our knowledge, this study is the first genome-wide report of genetic alterations in intramucosal adenocarcinoma. Although multiple genetic events accompanying the loss of genetic material caused by acquisition of LOH are required for tumour progression, our results suggest that gain of multiple chromosomal loci rather than genetic losses contributes to early colorectal carcinogenesis. Therefore, we suggest that molecular alterations may be characterized by chromosomal gains, which cause genomic instability as a result of DNA aneuploidy.

In a previous study, we examined genome-wide CNAs in advanced CRCs [22]. There were numerous CNAs in advanced CRCs in our previous study [22], and regions of gain detected in more than 50% of cases were located at 18q21.2-22.3 18q11.1-12.3 and 18q11.21-11.32. Although regions of LOH detected in more than 50% of cases were found at 18q21.2-22.3, 18q23,and 18q12.1-12.3, no regions with copy-neutral LOH in more than 50% of cases were detected [22]. Our findings are similar to those of previous reports in terms of the occurrence of multiple genomic changes in invasive CRC [24, 25]. These results indicate that there are several well-defined CNAs, including gains at 1q, 7p/q, 8p/p, 12q, 13q, 19q and 20p/q, and losses at 18p/q and 17p/q [25]. In addition, significant genomic losses were observed at 1p, 4q, 5q, 8p, 14q, 15q, 20p and 22q [25]. Another study investigated genomic changes in primary CRC and metastatic lesions [13], and revealed a characteristic pattern of CNAs in metastatic lesions from CRC that involved losses of regions at 1p, 17p, and 18q and gains of regions at 7 and 13q [13]. These findings suggest that acquisition of increasing genomic changes (CNAs) occurring in the tumour cell plays a major role in the progression or metastasis of CRC. From the results taken together, although we found that high levels of gains in intramucosal adenocarcinomas were detected at 13q, 7q, 8p, 20q, 7p, 18p and 17p in the present study, gains at 13q were common genomic changes in advanced CRC.

The most striking difference in chromosomal alterations between high-grade colorectal adenoma and intramucosal adenocarcinoma was an increase in the number of specific regions of gain, as detected with a high-resolution SNP array. The significance of this result in terms of tumour pathogenesis is not yet clear. In the present study, we showed that gains at 13q, 17p and 18p might play an essential role in early colorectal carcinogenesis. In particular, we suggest that gain at 13q is closely associated with progression to intramucosal adenocarcinoma from high-grade colorectal adenoma. We also searched for specific genes associated with colorectal carcinogenesis. If the regions at specific loci that we examined contribute to tumour progression, the product of the candidate gene is expected to be overexpressed in a given tissue. Although we could not find minimal common regions of CNAs (more than 30% of cases) in colorectal adenomas and intramucosal adenocarcinomas in the present study, we propose that three candidate genes—*FGF9* (which encodes fibroblast growth factor 9), *FLT1* (which encodes vascular endothelial growth factor receptor 1, also known as Fms-like tyrosine kinase 1) and *KLF5* (which encodes Krüppel-like factor 5), located at 13q11-12, 13q12 and 13q22.1 respectively—may be involved in tumour progression, in agreement with previously published studies (*FGF9* [26, 27], *FLT1* [28, 29] and *KLF5* [28–32]). Although these genes are found to be overexpressed in several malignant tumours, including gastric and colon cancers, it remains unclear whether the products of *FGF9*, *FLT1* and *KLF5* are overexpressed in colorectal intramucosal adenocarcinoma [26–28, 31]. Further examination of the expression of these genes is required.

The total length of CNAs is expected to be one of the factors closely associated with the level of genetic instability in tumour cells [33]. Although DNA aneuploidy is a classic factor representing chromosomal instability in tumour cells [34], the total length of CNAs may become a novel factor for identification of chromosomal instability of tumour cells [35]. In the present study, there were significant differences in the total lengths of overall CNAs and copy number gain between low-grade and high-grade colorectal adenomas. However, the significance of differences in the total length of copy number LOH between them was low. This finding suggests that although high-grade colorectal adenoma seems to be adenomatous histologically, such cells may acquire a higher level of chromosome instability at the high-grade lesion stage. On the other hand, no significant differences in the total lengths of CNAs (overall, LOH, copy-neutral LOH or gain) were found between high-grade adenoma and intramucosal adenocarcinoma, in contrast to the comparison of low-grade adenoma with high-grade adenoma. This finding suggests that although the histological difference between high-grade adenoma and intramucosal adenocarcinoma is clear to some extent, the molecular alterations in high-grade adenoma are similar to those in intramucosal adenocarcinoma at the level of chromosomal instability [16, 17]. In addition, this finding is highly useful to predict aggressive behaviour in terms of transformation to a more high-grade tumour from a low-grade tumour [36].

The Cancer Genome Atlas (TCGA) has identified comprehensive genetic alterations of various malignant tumours, including colorectal tumours [24]. Tumour genome data obtained from TCGA is considered standard genetic data worldwide. However, we do not think TCGA data are directly comparable with data of other studies given that the TCGA platform is different from that of other studies, including our own. In addition, genetic alterations in colorectal adenoma and intramucosal adenocarcinoma from TCGA data have not been fully clarified. We believe that our results contribute to the understanding of colorectal tumorigenesis.

According to previous studies, there are major differences in CRC when it occurs on the right side compared with the left side [22, 37, 38]. Previous studies showed that classification based on tumour location (i.e., left-sided CRC and right-sided CRC) is also useful and essential for evaluation of colorectal carcinogenesis [22, 37, 38]. In the present study, although we examined CNAs of low-grade adenoma, high-grade adenoma, and intramucosal adenocarcinoma for left-sided and right-sided tumours, no significant differences between them were found. Thus, because we did not identify distinct differences between left-sided and right-sided tumours, further examinations are required.

Previous studies suggested that the development of rectal and colonic cancers may involve different mechanisms [39, 40]. We examined CNAs of low-grade adenoma, high-grade adenoma, and intramucosal adenocarcinoma of the rectum and the colon. Although there were no differences in the frequency of CNAs between the rectum and the colon in low-grade adenoma, a significant difference in the frequency of CNAs between the rectum and the colon was observed for high-grade adenoma (gains at 8q23.2-3, 8p11.1 and p11.21-23.3). Moreover, there were significant differences in the frequencies of CNAs between the rectum and the colon for intramucosal adenocarcinomas (greater for the rectum than for the colon: gains at 16q22.1-24.3, 21q21.3-22.3, 10q11.21-23, 10p11.1, 12q13.13-15, q21.2, q24.32, 12p11.1-13.31, 16q11.2-13 and 21q11.2-21.2; LOH at 16p13.3). These data along with existing evidence for the presence of distinct genetic profiles may be supportive of the concept that rectal and colonic CRCs are distinct molecular entities [39, 40]. However, the conclusions derived from our findings are limited because of the small number of cases, and thus further studies with greater numbers of cases are needed.

Although elucidation of the molecular differences between adenoma and intramucosal adenocarcinoma within the same tumour may be needed to evaluate the early stages of colorectal carcinogenesis, it is difficult to evaluate differences between lesions within an individual tumour. In fact, distinguishing adenomatous lesions (in particular, high-grade adenoma) from intracarcinomatous lesions on the basis of surface macroscopic findings is very difficult. We will examine differences in genome-wide alterations within the same tumour in future studies.

In conclusion, we attempted to better define the molecular mechanism that initiates chromosomal instability during colorectal tumorigenesis. Identification of the relationship between chromosomal instability and tumour progression and the feasibility of targeting chromosomally unstable cells will be effective in advancing our understanding of tumour characteristics during the initial phases of colorectal tumorigenesis.

## Electronic supplementary material

Below is the link to the electronic supplementary material.

Supplementary material 1 (DOCX 81 kb)

Supplementary material 2 (DOCX 72 kb)

## Abbreviations

BAF: B allele frequency
CNA: Copy number alteration
CNLOH: Copy-neutral loss of heterozygosity
CRC: Colorectal cancer
LOH: Loss of heterozygosity
LRR: Log *R* ratio
SNP: Single-nucleotide polymorphism
