# Supplementary material for: Molecular alterations in colorectal adenomas and intramucosal adenocarcinomas defined by high-density single-nucleotide polymorphism arrays
Source: J Gastroenterol. 2017 Feb 14;52(11):1158–68. doi: 10.1007/s00535-017-1317-2 (PMC5666076; doi:10.1007/s00535-017-1317-2)
Supplement: Supplementary file 1 — Supplementary material 1 (DOCX 81 kb) [file 535_2017_1317_MOESM1_ESM.docx]

Supplementary Table 1: Genomic differences of low- and high-grade colorectal adenomas and high- grade colorectal adenoma and intramucosal adenocarcinomas between left-sided and right-sided tumors

| **Chromosomal regions** | Left-sided LGA  n =20 (%) |  | Right-sided LGA  n=15 (%) |  | *P*-value |
| --- | --- | --- | --- | --- | --- |
| **Gain** |  |  |  |  |  |
| 7q21.11-21.3 | 5 (25.0) |  | 0 |  | 0.11 |
| 7p21.1-3 | 5 (25.0) |  | 0 |  | 0.11 |
| 7q22.2-31.33,7p11.2-12.1,7p15.3 | 4 (20.0) |  | 0 |  | 0.19 |
| **CNLOH** |  |  |  |  |  |
| 3q13.2-33 | 0 |  | 2 (13.3) |  | 0.34 |
| 22q12.2-3 | 0 |  | 2 (13.3) |  | 0.34 |
| 1q41-42.2 | 1 (5.0) |  | 1 (6.7) |  | 0.60 |
| 2q36.3-37.1 | 1 (5.0) |  | 1 (6.7) |  | 0.60 |
| 3q22.3-37.1 | 1 (5.0) |  | 1 (6.7) |  | 0.60 |
| **LOH** |  |  |  |  |  |
| 16p13.3 | 0 |  | 2 (13.3) |  | 0.34 |
| 1p35.3-36.22, 5q22.3, 6q26 | 1 (5.0) |  | 1 (6.7) |  | 0.60 |
| 1p36.23-31 | 1 (5.0) |  | 2 (13.3) |  | 0.79 |
|  |  |  |  |  |  |
| **Chromosomal regions** | Left-sided HGA  n =15 (%) |  | Right-sided HGA  n=5 (%) |  | *P*-value |
| **Gain** |  |  |  |  |  |
| 6q11.1, 6p11.1-25.3 | 4 (26.7) |  | 0 |  | 0.52 |
| 12q11-24.33, 12p11.1-13.31 | 4 (26.7) |  | 0 |  | 0.52 |
| 20q11.21-13.33 | 4 (26.7) |  | 0 |  | 0.52 |
| **CNLOH** |  |  |  |  |  |
| 16p12.1 | 1 (6.7) |  | 2 (40.0) |  | 0.28 |
| 5q14.3 | 4 (26.7) |  | 3 (60.0) |  | 0.42 |
| 3p25.1-26.3 | 3 (20.0) |  | 1 (20.0) |  | 0.52 |
| 5q13.3 | 2 (13.3) |  | 2 (40.0) |  | 0.52 |
| **LOH** |  |  |  |  |  |
| 1p36.12-32 | 0 |  | 2 (40.0) |  | 0.09 |
| 16p13.3 | 2 (13.3) |  | 2 (40.0) |  | 0.52 |
| 1q42.2-3 | 0 |  | 1 (20.0) |  | 0.55 |
| 1p21.1-21.3 | 1 (6.7) |  | 0 |  | 0.55 |
|  |  |  |  |  |  |
| **Chromosomal regions** | Left-sided IMA  n =21 (%) |  | Right-sided IMA  n=9 (%) |  | *P*-value |
| **Gain** |  |  |  |  |  |
| 15q21.3-22.2 | 1 (4.8) |  | 3 (33.3) |  | 0.13 |
| 4q12-35.2, 4p16.2-3 | 0 |  | 2 (22.2) |  | 0.15 |
| 14q11.2, q13.2, q32.12-13 | 0 |  | 2 (22.2) |  | 0.15 |
| **CNLOH** |  |  |  |  |  |
| 17p11.2-12, p13.2 | 4 (19.0) |  | 0 |  | 0.41 |
| 2p11.2-12 | 1 (4.8) |  | 2 (22.2) |  | 0.43 |
| 5q12.1-13.1 | 2 (9.5) |  | 1 (11.1) |  | 0.60 |
| 6p21.1 | 3 (14.3) |  | 0 |  | 0.60 |
| **LOH** |  |  |  |  |  |
| 18q21.2, q21.32 | 6 (28.6) |  | 0 |  | 0.20 |
| 18q12.3-21.1, q21.31, q21.33-23 | 5 (23.8) |  | 0 |  | 0.29 |
| 8p12-23.2 | 4 (19.0) |  | 0 |  | 0.41 |
| 18q11.2-12.1, 18p11.21-22 | 4 (19.0) |  | 0 |  | 0.41 |
|  |  |  |  |  |  |

LGA, low grade adenoma; HGA, high grade adenoma; IMA, intramucosal adenocarcinoma; CNLOH, copy neutral loss of heterozygosity
