# Supplementary material for: Molecular alterations in colorectal adenomas and intramucosal adenocarcinomas defined by high-density single-nucleotide polymorphism arrays
Source: J Gastroenterol. 2017 Feb 14;52(11):1158–68. doi: 10.1007/s00535-017-1317-2 (PMC5666076; doi:10.1007/s00535-017-1317-2)
Supplement: Supplementary file 2 — Supplementary material 2 (DOCX 72 kb) [file 535_2017_1317_MOESM2_ESM.docx]

Supplementary Table 2: Genomic differences of low- and high-grade colorectal adenomas and high- grade colorectal adenoma and intramucosal adenocarcinomas between rectal and colonic tumors

| chromosomal regions | Colonic HGA  n=15 (%) | Rectal HGA  n=5 (%) | *P*-value |
| --- | --- | --- | --- |
| **Gain** |  |  |  |
| 8q23.2-3, 8p11.1, p11.21-23.3 | 0 | 3 (60.0) | <0.01 |
| **CNLOH** | None |  |  |
| **LOH** | None |  |  |

| chromosomal regions | Colonic IMA  n=23 (%) | Rectal IMA  n=7 (%) | *P*-value |
| --- | --- | --- | --- |
| **Gain** |  |  |  |
| 16q22.1-24.3 | 0 | 3 (42.9) | <0.01 |
| 21q21.3-22.3 | 0 | 3 (42.9) | <0.05 |
| 10q11.21-23, 10p11.1 | 1 (4.3) | 3 (42.9) | <0.05 |
| 12q13.13-15, q21.2, q24.32 | 1 (4.3) | 3 (42.9) | <0.05 |
| 12p11.1-13.31 | 1 (4.3) | 3 (42.9) | <0.05 |
| 16q11.2-13 | 1 (4.3) | 3 (42.9) | <0.05 |
| 21q11.2-21.2 | 1 (4.3) | 3 (42.9) | <0.05 |
| **CNLOH** | None |  |  |
| **LOH** |  |  |  |
| 16p13.3 | 0 | 3 (42.9) | <0.01 |

LGA, low grade adenoma; HGA, high grade adenoma; IMA, intramucosal adenocarcinoma, CNLOH, copy neutral loss of heterozygosity
